# Genomic Evaluation of *Thermoanaerobacter* spp. for the Construction of Designer Co-Cultures to Improve Lignocellulosic Biofuel Production

**DOI:** 10.1371/journal.pone.0059362

**Published:** 2013-03-26

**Authors:** Tobin J. Verbeke, Xiangli Zhang, Bernard Henrissat, Vic Spicer, Thomas Rydzak, Oleg V. Krokhin, Brian Fristensky, David B. Levin, Richard Sparling

**Affiliations:** 1 Department of Microbiology, University of Manitoba, Winnipeg, Manitoba, Canada; 2 Department of Plant Science, University of Manitoba, Winnipeg, Manitoba, Canada; 3 Centre national de la recherche scientifique, Aix-Marseille Université, Marseille, France; 4 Department of Physics & Astronomy, University of Manitoba, Winnipeg, Manitoba, Canada; 5 Department of Internal Medicine & Manitoba Centre for Proteomics and Systems Biology, University of Manitoba, Winnipeg, Manitoba, Canada; 6 Biosystems Engineering, University of Manitoba, Winnipeg, Manitoba, Canada; INRA Clermont-Ferrand Research Center, France

## Abstract

The microbial production of ethanol from lignocellulosic biomass is a multi-component process that involves biomass hydrolysis, carbohydrate transport and utilization, and finally, the production of ethanol. Strains of the genus *Thermoanaerobacter* have been studied for decades due to their innate abilities to produce comparatively high ethanol yields from hemicellulose constituent sugars. However, their inability to hydrolyze cellulose, limits their usefulness in lignocellulosic biofuel production. As such, co-culturing *Thermoanaerobacter* spp. with cellulolytic organisms is a plausible approach to improving lignocellulose conversion efficiencies and yields of biofuels. To evaluate native lignocellulosic ethanol production capacities relative to competing fermentative end-products, comparative genomic analysis of 11 sequenced *Thermoanaerobacter* strains, including a *de novo* genome, *Thermoanaerobacter thermohydrosulfuricus* WC1, was conducted. Analysis was specifically focused on the genomic potential for each strain to address all aspects of ethanol production mentioned through a consolidated bioprocessing approach. Whole genome functional annotation analysis identified three distinct clades within the genus. The genomes of Clade 1 strains encode the fewest extracellular carbohydrate active enzymes and also show the least diversity in terms of lignocellulose relevant carbohydrate utilization pathways. However, these same strains reportedly are capable of directing a higher proportion of their total carbon flux towards ethanol, rather than non-biofuel end-products, than other *Thermoanaerobacter* strains. Strains in Clade 2 show the greatest diversity in terms of lignocellulose hydrolysis and utilization, but proportionately produce more non-ethanol end-products than Clade 1 strains. Strains in Clade 3, in which *T. thermohydrosulfuricus* WC1 is included, show mid-range potential for lignocellulose hydrolysis and utilization, but also exhibit extensive divergence from both Clade 1 and Clade 2 strains in terms of cellular energetics. The potential implications regarding strain selection and suitability for industrial ethanol production through a consolidated bioprocessing co-culturing approach are examined throughout the manuscript.

## Introduction

Consolidated bioprocessing (CBP), whereby microbial enzyme production, biomass hydrolysis and substrate conversion to ethanol all occurs in a single bioreactor, offers improved economic feasibility and process efficiencies compared with alternative approaches to lignocellulosic ethanol production [1]–[3]. However, to date, no single organism has been identified that is capable of performing all of these tasks at industrially significant levels [4]. The utilization of designer co-cultures, allowing for potential complementary or synergistic phenotypes between multiple organisms to improve process efficiencies and yields, is an alternative strategy to potentially overcome these limitations (reviewed by Brenner *et al*. [5] and Zuroff & Curtis [6]).

From a CBP standpoint, the use of thermophilic, anaerobes belonging to the Firmicutes offers many advantages. Growth at elevated temperatures reduces energy costs by avoiding repeated heating and cooling steps associated with cycling between microbial growth and both upstream pre-processing as well as downstream product recovery. Additionally, the native capacity for many strains to produce lignocellulose hydrolytic enzymes *in situ* may reduce or eliminate the need for enzymatic pre-treatment of biomass. Finally, the ability of multiple strains to ferment a broad range of lignocellulose constituent saccharides into ethanol allows for efficient conversion and utilization of the biomass.

To date, the organisms garnering the most attention for thermophilic CBP include strains in the orders *Clostridiales* or *Thermoanaerobacteriales* [7]. One of these organisms, *Clostridium thermocellum,* has been at the forefront of lignocellulosic ethanol research for decades due to its high cellulolytic capabilities [8]–[10]. However, the inability of *C. thermocellum* to ferment pentose sugars resulting from hemicellulose hydrolysis reduces potential biomass conversion efficiencies and represents a major limitation in its development as an industrial microorganism. Various strains of the genus *Thermoanaerobacter,* on the other hand, are known to hydrolyze hemicellulose and ferment hemicellulose constituent sugars [11]–[15], naturally produce [11], [16] and tolerate [13], [17] comparatively high ethanol concentrations, and are amenable to genetic manipulation for purposes of further improving biofuel yields [18], [19]. Furthermore, previous studies have reported that cellulose degradation rates and overall biofuel yields are improved by *C. thermocellum*-*Thermoanaerobacter* spp. co-cultures as compared to *C. thermocellum* mono-cultures [20]–[23]. As such, the use of a *Thermoanaerobacter* strain as an industrially relevant co-culture partner for *C. thermocellum* shows great potential as a CBP strategy.

**Figure 1:**
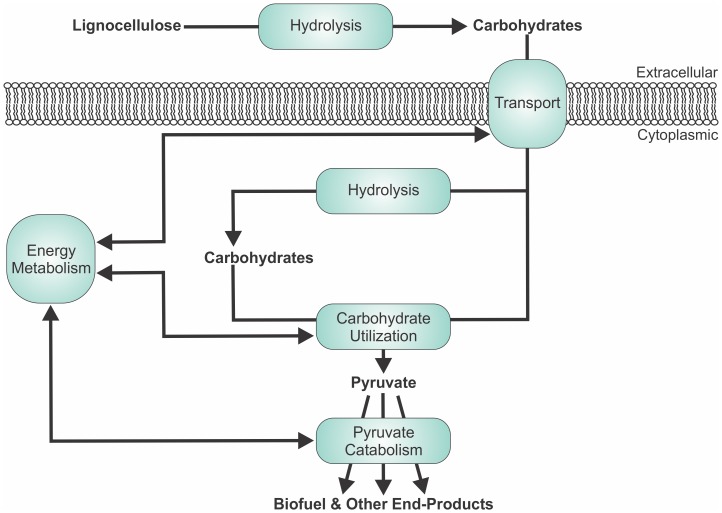
Schematic representation of key physiological processes pertinent to lignocellulosic ethanol production in a CBP system. Physiological processes are identified as the text in blue boxes.

The genomes of multiple *Thermoanaerobacter* spp. are currently available publicly. The purpose of the current publication is to conduct genus wide comparative genomic analysis of all available genomes, including a *de novo* genome from *Thermoanaerobacter thermohydrosulfuricus* WC1 [15], [24], and evaluate the potential of each strain as a *C. thermocellum* co-culture partner. Factors evaluated include the capacity for lignocellulose hydrolysis, transport of the resulting hydrolysis products, carbohydrate utilization and the potential to produce ethanol relative to other fermentation end-products. Further, given that these processes are interconnected with cellular energy metabolism (Figure 1), the potential mechanisms for energy conservation will also be evaluated.

## Methods

### DNA Extraction, Genome Sequencing and Assembly

In lab glycerol stock cultures of *T. thermohydrosulfuricus* WC1 were revived, grown overnight and plated as previously described [15]. A single colony was picked and used to inoculate fresh ATCC 1191 liquid medium (10 mL) containing 2 g L^−1^ cellobiose. The resulting culture was allowed to grow overnight and gDNA was extracted using the Wizard® Genomic DNA Purification kit (Promega Corp, Madison, WI).

The genome of *T. thermohydrosulfuricus* WC1 was first sequenced at the McGill University and Genome Quebec Innovation Centre using shotgun 454 pyrosequencing [25] to get 121,690 reads with an average length of 626 bp. Genome assembly by Newbler v2.6 generated 123 contigs (size >100 bp) with the longest contig 182,175 bp with ∼15× depth coverage. To improve gap closure and perform scaffolding of the contigs, genome re-sequencing was conducted with Illumina HiSeq 2000 paired-end technologies generating 122,042,203 reads of 100 bp from each end with an average insert size of 277 bp. Illumina reads were pre-processed by adaptor clipping, quality trimming with the FASTX-Toolkit [26] and random subset data selection. Multiple assembly pipelines combining both 454 and Illumina data were evaluated including Optimized-Velvet [27], Ray [28] and Newbler v.2.6. Based on statistics metrics, Newbler v2.6 generated the longest contigs with the highest N50 and the resulting assembly was composed of 47 scaffolds plus an additional 15 unscaffolded contigs (size >100 bp with the longest contig 261,431 bp) with ∼56× depth coverage.

### Genome Annotation and Proteogenomic Analysis

The assembled DNA scaffolds and contigs were submitted to the Joint Genome Institute’s (JGI) Integrated Microbial Genomes-Expert Review (IMG-ER) platform [29] for gene calling and annotation using their annotation pipeline (http://img.jgi.doe.gov/w/doc/img_er_ann.pdf). The annotated genome was subsequently submitted to the JGI’s GenePRIMP pipeline [30], which reported 358 anomalies. All anomalies were manually curated.

Proteogenomic analysis provided supporting evidence for manual curation decisions regarding reported GenePRIMP anomalies. A mechanism of genome assembly independent proteomics, similar to the approach by Krug *et al*. [31], was implemented for this analysis. In brief, raw genomic sequencing reads for *T. thermohydrosulfuricus* WC1, rather than an assembled genome, were transcribed into amino acid sequences in all six possible reading frames with all potential STOP codons being reported as new elements. These elements were subjected to *in silico* tryptic digestion to create a “naïve” peptide database. This database was used to search a 2D-HPLC-MS/MS experimental output, generated from mid-exponential phase *T. thermohydrosulfuricus* WC1 cells grown in liquid medium (as described above), using a high-performance GPU-based identification engine developed for this project [32]. Correlating observed peptide retention times against their computed hydrophobicities as described by Krokhin [33] further supported the assignment confidences. As the resultant *in silico* naïve peptide collection was wholly disconnected from their source proteins, the peptide database served as a validation for the genome assembly and annotation workflows. Observed peptides were compared against the annotation-derived protein database and those unique to the “naïve” database search were reported. Unique peptides (869), corresponding to unannotated protein coding sequences (CDS) within the genome, were used to support or refute GenePRIMP identified annotation anomalies. Modifications to the annotation based upon these peptide data are reported as “notes” within the GenBank file associated with the genome. The genome has been deposited at DDBJ/EMBL/GenBank under accession number AMYG00000000. The version described in this paper is the first version, AMYG01000000.

### Comparative Genomic Analysis

Comparative genomic analysis was conducted on genomes and gene annotations available (as of July 2012) using the IMG-ER platform unless specified elsewhere. Genes of interest, with the exception of transporters and carbohydrate active enzymes (CAZymes), were identified within the IMG-ER annotated genomes using independent searches for the Clusters of Orthologous Groups (COG) [34], KEGG Orthology (KO) [35] and TIGRFAMs [36] unique identifiers. Transporters were identified using the assignment criteria given by the Transporter Classification Database (TCDB) [37] as part of the IMG-ER system and substrate specificities were inferred based upon KO annotations of the same genes. The annotation accuracy for all genes identified using the above search methods was manually assessed using a combination of genomic contextual analysis, sequence alignments and through literature and database searches. Gene annotations using additional databases made available through the IMG-ER annotation pipeline including Pfams [38], IMG Terms [29], the SEED [39] and Interpro [40] were used in the manual assessment of COG, KO and TIGRFAM designations when appropriate. Analysis of CAZymes was conducted by accessing the CAZy database [41] for all *Thermoanaerobacter* genomes available in the database or analysed *de novo*. For *Thermoanaerobacter ethanolicus* CCSD1 and *Thermoanaerobacter* sp. X561 the unfinished draft genomes were downloaded from the NCBI and were analyzed using the standard detection pipeline of the CAZy database. Substrate specificities for all CAZymes were not inferred unless specifically discussed (in Results & Discussion) and were instead limited to substrates reported for each enzyme class by the CAZy database. The subcellular localization of identified CAZymes was predicted by uploading fasta files of all identified gene amino acid sequences into the PSORTb 3.0 database [42] and using the final predictions.

### Sequence and Phylogenetic Analysis

Sequence alignments were performed using BioEdit v.7.0.9.0 [43]. Phylogenetic analysis of individual sequences was performed using MEGA 4 [44]. Phylogeny was inferred using the neighbor-joining method with evolutionary distances calculated via the Poisson correction as described elsewhere [45]. Alignment gaps were deleted via pair wise sequence comparisons only and clusters grouped using the bootstrapping method (10,000 replicates).

## Results and Discussion

### Genome Properties of *T. thermohydrosulfuricus* WC1

The draft genome sequence of *T. thermohydrosulfuricus* WC1 is comprised of 2, 573, 514 bp and shows a G+C content of 34.35%, which is consistent with other sequenced *Thermoanaerobacter* strains (Table S1). There are 2,655 genes annotated with 2,552 predicted to be CDS. The chaperonin-60 universal target (*cpn*60 UT) nucleotide sequence, which has been shown to be a more reliable phylogenetic indicator than 16S rRNA encoding DNA for the *Thermoanaerobacter* genus [24], agrees 100% with the previously published sequence [15]. Based on the standards for genomic sequencing projects [46], the *T. thermohydrosulfuricus* WC1 permanent draft-genome belongs to the “Annotation-Directed Improvement” classification.

### Whole Genome Comparative Analysis

The genomes of 11 *Thermoanaerobacter* strains (10 publicly available as of July 2012+ *T. thermohydrosulfuricus* WC1) were included in our genus wide comparison. The *Thermoanaerobacter tengcongensis* MB4 genome [47], corresponding to GenBank accession number: AE008691 was not included based upon the reclassification of the strain into the genus *Caldanaerobacter* [48]. The available genome sequences for the genus, in draft, permanent draft and finished states, range in size from 2.20 Mb –2.78 Mb and are annotated to contain anywhere from 2286–2800 CDS (Table S1).

**Figure 2:**
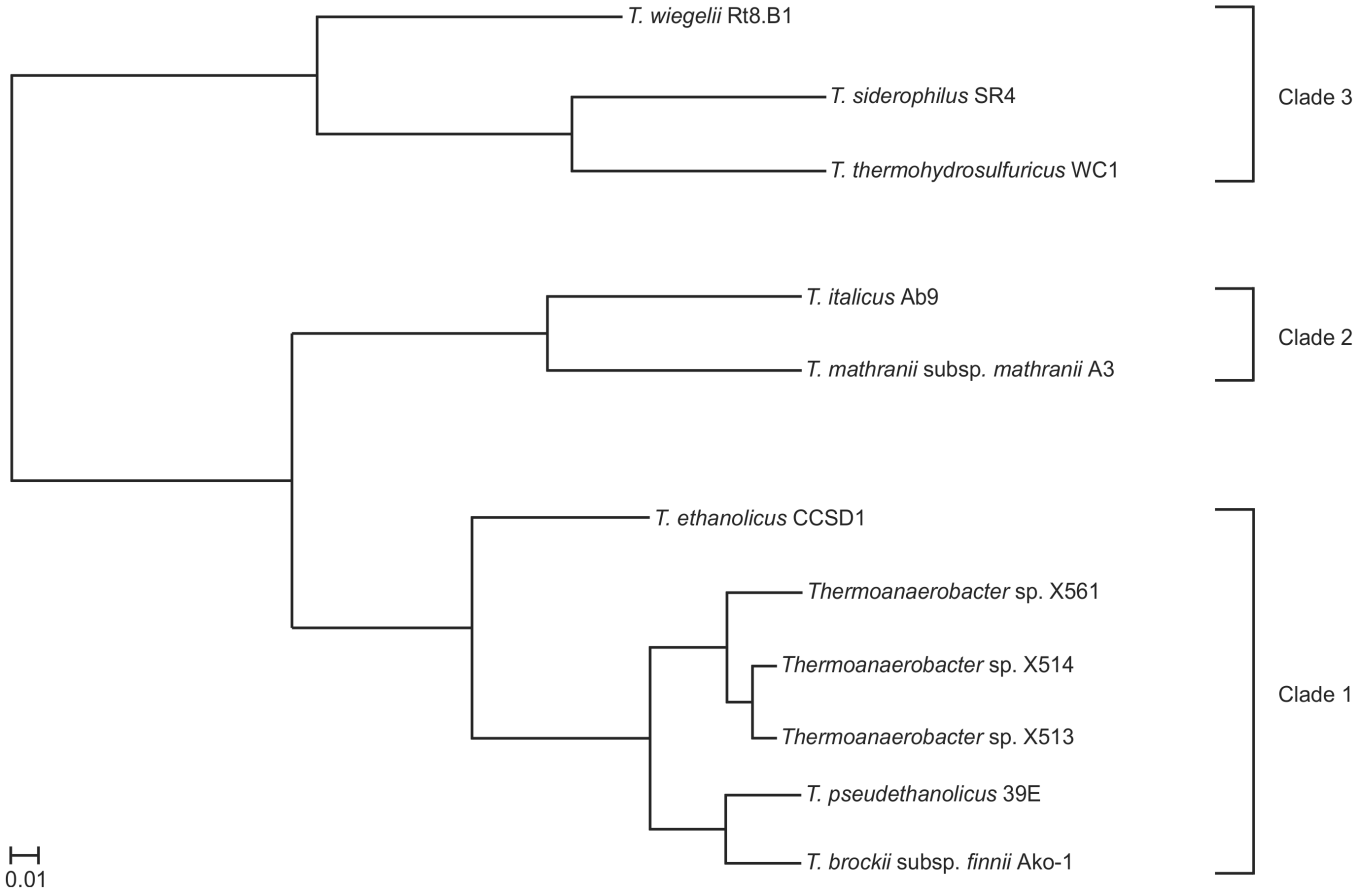
Phylogram of annotated COG functional profiles for sequenced *Thermoanaerobacter* strains. Cluster analysis, based on Cluster 3.0 analysis software [120], was conducted within the IMG-ER platform using the COG profiles for each genome. Branch lengths correspond to calculated distances between functional profiles. Similar clade architectures are observed when using KO or TIGRFAM descriptors (not shown).

To determine the extent that similar functional profiles exist between strains, hierarchical clustering, based upon COG, KO and TIGRFAM qualifiers, was conducted independently for each qualifier. As shown in Figure 2, three distinct clades exist within the genus. Clade 1 contains the most genomes and could also potentially be divided into sub-clades. However, the study by Verbeke *et al*. [24] determined that all strains in Clade 1 share an average nucleotide identity (ANIm) score [49] greater than 0.97 and thus, may represent strains of the same species. Previous work by Hemme *et al*. [23] with *Thermoanaerobacter pseudethanolicus* 39E (formerly called *Clostridium thermohydrosulfuricum* 39E [16] and *Thermoanaerobacter ethanolicus* 39E [50]) and *Thermoanaerobacter* sp. X514, have shown that genomes with ANIm scores greater than 0.97 may have significant differences that impact relative strain suitability for lignocellulosic ethanol production. Thus, inter-clade functional divergence may have an even more drastic impact on co-culturing potential than intra-clade divergence. Specifics regarding strain suitability are discussed below.

### CAZyme Analysis

The availability of fermentable sugars from lignocellulosic biomass is dependent on CAZymes capable of degrading the insoluble extracellular carbohydrate polymers (Figure 1). In designer co-cultures, the multiple hemicellulase enzymes in the *C. thermocellum* cellulosome may help to liberate hemicellulose constituent sugars from the polymeric backbone. The liberation of such saccharides, most notably pentoses, but also a mixture of hexoses, may subsequently be fermented to ethanol by a *Thermoanaerobacter* strain. However, expression analysis studies of *C. thermocellum* grown on various substrates by Raman *et al*. [10] have shown that xylanase expression levels were at their lowest when grown on a hemicellulose containing substrate (switchgrass) in comparison to growth on cellobiose or cellulose. Thus, under CBP conditions, the utilization of a *Thermoanaerobacter* strain which has xylan hydrolysis capabilities may help facilitate biomass hydrolysis similar to what has been reported in co-cultures of *C. thermocellum* and *Caldicellulosiruptor bescii* grown on switchgrass or cellulose+xylan [51].

To examine the potential that a *Thermoanaerobacter* strain may contribute to biomass hydrolysis, all CAZyme genes were identified within the genus. As shown in Table S2, between 45–61 independent CAZyme gene sequences, belonging to 27–40 distinct CAZyme classes, were identified within the sequenced strains.

A small subset (GH23 and various glycosyltransferases) of these genes encode proteins typically associated with cellular maintenance functions such as peptidoglycan processing and glycogen synthesis. Furthermore, based on the PSORTB 3.0 predictions, only a small fraction of each CAZyome is predicted to be localized extracellularly (Table 1) such that only a few genes per strain would be capable of contributing to the extracellular hydrolysis of complex polymers.

**Table 1:** Predicted extracellular CAZymes involved with lignocellulosic biomass hydrolysis within sequenced *Thermoanaerobacter* spp.

	Clade 1						Clade 2		Clade 3		
CAZyme Designation&ModularStructure	*T. brockii*subsp.*finnii*Ako-1	*T. ethanolicus*CCSD1	*T. pseudethanolicus*39E	*Thermoanaerobacter* sp. X513	*Thermoanaerobacter* sp. X514	*Thermoanaerobacter* sp. X561	*T. italicus*Ab9	*T. mathranii*subsp.*Mathranii*A3	*T. siderophilus*SR4	*T. thermohydrosulfuricus*WC1	*T. wiegelii*Rt8.B1
GH10							Thit_0188	Tmath_0247			
GH43								Tmath_1695			
GH52							Thit_0190	Tmath_0249		TthWC1_1012	
CE4									ThesiDRAFT1_1967	TthWC1_1808	Thewi_00017610
PL9							Thit_1727				
GH66-CBM35-CBM35-GH15									ThesiDRAFT1_0902	TthWC1_0529	
CBM22-CBM22-GH10-CBM9-CBM9-SLH-SLH-SLH							Thit_0192	Tmath_0251		TthWC1_1010	

#### Extracellular, lignocellulose hydrolyzing CAZymes

As is shown in Table 1, no predicted extracellular lignocellulose hydrolyzing CAZymes were identified in Clade 1 strains. Moreover, only 3 strains, *T. italicus* Ab9, *T. mathranii* subsp. *mathranii* A3 and *T. thermohydrosulfuricus* WC1, possess extracellular endo-xylanases capable of hydrolyzing the xylan backbone of many hemicelluloses. All three strains contain a secretable multi-component CDS with the modular structure CBM22-CBM22-GH10-CBM9-CBM9-SLH-SLH-SLH. To date, proteins belonging to the GH10 family are only known to act on xylan, while CBM22 and CBM9 modules are considered to be primarily xylan binding. All orthologs show high amino acid sequence similarity (>74%) and similar modular structure to the functionally characterized *xyn*A gene in *Thermoanaerobacterium saccharolyticum* NTOU1 [52].

*T. italicus* Ab9 (Thit_0188) and *T. mathranii* subsp. *mathranii* A3 (Tmath_0247) also contain an additional extracellular GH10 family gene showing high amino acid sequence similarity (>77%) with the cloned and characterized xylanase/β-xylosidase from *Caldicellulosiruptor saccharolyticus* DSM 8903 [53].

The hydrolysis products resulting from GH10 mediated endo-xylanase activity would be xylo-oligomers. The generation of xylose monomers from xylo-oligomers requires extracellular and/or intracellular (see below) β-xylosidase activity. The *T. italicus* Ab9, *T. mathranii* subsp. *mathranii* A3 and *T. thermohydrosulfuricus* WC1 genomes also encode for an extracellular GH52 family enzyme (Table 1, Table S2), which, to date, are solely reported to have β-xylosidase activity. Additionally, unique to *T. mathranii* subsp. *mathranii* A3 is a putatively cell bound GH43 enzyme, which may have further pentose releasing hydrolysis capabilities.

The identification that only three of the strains evaluated contain GH10 family enzymes is consistent with the reports that only *T. italicus* Ab9 [12], *T. mathranii* subsp. *mathranii* A3 [13] and *T. thermohydrosulfuricus* WC1 (our lab, unpublished results) are capable of growing on xylan polymers. Other sequenced strains lacking extracellular xylanolytic enzymes, specifically *T. siderophilus* SR4 [54], *Thermoanaerobacter* sp. X514 [23] and *T. pseudethanolicus* 39E [20], have been specifically reported to not grow on xylan. The ability to use, or not use, xylan by the remaining *Thermoanaerobacter* strains has not yet been reported.

*T. italicus* Ab9 has the sole genome containing a pectate lyase [12], while genomes of *T. thermohydrosulfuricus* WC1 and *T. wiegelii* Rt8.B1 are the only two that encode a putative extracellular acetyl-xylan esterase for removal of acetyl-groups from xylan polymers. A final unique extracellular enzyme found in *T. thermohydrosulfuricus* WC1 and *T. siderophilus* SR4 (TthWC1_0529; ThesiDRAFT1_0902) has a modular structure of GH66-CBM35-CBM35-GH15. No database (GenBank – non redundant protein) entry yet is homologous over the entire length of these CDS, as <60% query coverage is observed when comparing all database entries with either the TthWC1_0529 or ThesiDRAFT1_0902 queries. Reported activities associated with GH66 and GH15 family proteins are reported to act on α-glucan linkages, while CBM35 modules reportedly bind β-linkages (xylans, mannans, galactans), but the functional role of these enzymes is unknown.

#### Intracellular, characterized lignocellulose hydrolyzing CAZymes

Only a few glycoside hydrolases with lignocellulose hydrolysis potential have been functionally characterized in any *Thermoanaerobacter* strain and these are all predicted to be localized intracellularly. One of these, a GH3 family protein *xarB* from *T. ethanolicus* JW200, was initially cloned and characterized and was reported to cleave β-1,4-xylobiose linkages as well as remove arabinosyl groups from xylo-oligomers [55]. Orthologous sequences, showing conserved genomic organization to *T. ethanolicus* JW200, are found in *T. italicus* Ab9 (Thit_0198), *T. mathranii* subsp. *mathranii* A3 (Tmath_0257) and *T. thermohydrosulfuricus* WC1 (TthWC1_1005) (also see Figure 6 in [23]). Alternatively, a near identical, though N-terminally truncated *xarB* ortholog, *xglS*, was characterized in *T. brockii* subsp. *brockii* HTD4 [56] and also shown to have β-xylosidase activity. Similarly to *T. brockii* subsp. *brockii* HTD4, the *xglS* orthologs in *T. brockii* subsp. *finnii* Ako-1 (Thebr_2099) and *T. pseudethanolicus* 39E (Teth39_2055) sequences are co-localized with a *cglT* (GH1) ortholog (Thebr_2098; Teth39_2054) immediately downstream. The *xglS* ortholog in *T. wiegelii* Rt8.B1 (Thewi_00009950) is separated from its *cglT* ortholog (Thewi_00009970) by a hypothetical protein, while the GH3 enzymes of *Thermoanaerobacter* spp. X513, X514 and X561, as well as *T. siderophilus* SR4 do not show conserved genomic organization to the experimentally characterized *xarB* or *xglS* sequences.

The present analysis indicates that none of the GH3 CDS are predicted to be secreted extracellularly is in agreement with the findings for *T. thermohydrosulfuricus* JW 102 [55]. The predicted cytosolic location of this enzyme suggests that xylo-oligosaccharides, and not just xylose, are capable of being transported into the cell. The presence of both extracellular GH52 and cytosolic GH3 β-xylosidase orthologs in the *T. italicus* Ab9, *T. mathranii* subsp. *mathranii* A3 and *T. thermohydrosulfuricus* WC1 genomes is an interesting redundancy regarding xylan/xylo-dextrin hydrolysis patterns within these organisms, though the potential impact it may have on xylan hydrolysis and utilization in raw substrates is unknown.

The study by Breves *et al*. [56] also characterized a GH1 family protein, designated *cglT,* which is also predicted to be localized intracellularly. In *T. brockii* subsp. *brockii* HTD4, *cglT* is capable of hydrolyzing β-1,4-glucosidic linkages (up to cellopentaose tested) as well as the β-1,3- and β-1,2-glucosidic linkages of laminaribose and sophorose respectively and is co-localized with *xglS* (see above). Only three sequenced strains, *T. brockii* subsp. *finnii* Ako-1, *T. pseudethanolicus* 39E and *T. wiegelii* Rt8.B1 share similar genomic organization. GH1 domain containing sequences, highly similar to the *cglT* gene in *T. brockii* subsp. *brockii* HTD4, can be identified in all other *Thermoanaerobacter* strains, but are not co-localized with *xglS* and thus, may simply represent *cglT* homologs, rather than orthologs.

The release of fermentable sugars from lignocellulosic biomass is often considered the rate limiting step in simultaneous saccharification and fermentation processes [57]–[59]. Enzymatic pre-treatment of biomass is a common strategy for improving lignocellulose saccharification [60], though the accumulation of soluble sugars generated through hydrolysis are well known to have an inhibitory effect on continued enzymatic activity [61], [62]. Simultaneous incubation of exogenous enzymes and sugar fermenting bacteria is one strategy to relieve this inhibition and continue driving hydrolysis. However, Podkaminer *et al*. [63] have recently shown that in some cases, exogenous enzyme activity is reduced when incubated under conditions amenable for bacterial growth. Thus, using the hydrolytic machinery native to ethanol producing strains is favorable in terms of maintaining enzymatic activity and limiting costs associated with exogenous enzyme addition. The present analysis of the *Thermoanaerobacter* spp. genomes has identified that only *T. italicus* Ab9, *T. mathranii* subsp. *mathranii* A3 and *T. thermohydrosulfuricus* WC1 possess the potential enzymatic machinery needed to hydrolyze complex xylan polymers and potentially facilitate lignocellulose hydrolysis under CBP conditions.

### Carbohydrate Transport

The extracellular hydrolysis of insoluble carbohydrate polymers requires that the resulting soluble saccharides be transported into the cell prior to fermentation (Figure 1). Based upon genome annotations, *Thermoanaerobacter* spp. import carbohydrates via ABC-type transporters, phosphotransferase system (PTS) transporters and via cationic symporters (Table 2). Of the three annotated systems, ABC-type transporters, are the most abundant as *Thermoanaerobacter* spp. contain anywhere from 123–150 total genes belonging to the ATP-binding Cassette (ABC) Superfamily (TC:3.A.1) based upon TCDB designations. Only a subset of these will be involved with carbohydrate import, and to date, only a few of this subset have been characterized within any strain of the genus.

**Table 2:** Selected^1^ transporters associated with carbohydrate import identified within sequenced *Thermoanaerobacter* spp.

	Clade 1						Clade 2		Clade 3		
	*T. brockii* subsp. *finnii* Ako-1	*T. ethanolicus* CCSD1	*T. pseudethanolicus* 39E	*Thermoanaerobacter* sp. X513	*Thermoanaerobacter* sp. X514	*Thermoanaerobacter* sp. X561	*T. italicus* Ab9	*T. mathranii* subsp. *mathranii* A3	*T. siderophilus* SR4	*T. thermohydrosulfuricus* WC1	*T. wiegelii* Rt8.B1
**Cationic Symporters**											
2-Keto-3-Deoxygluconate	+	+	+	−	−	−	−	−	−	−	−
Glycoside/Pentoside/Hexuronide Cation Symporter	+	+	+	+	+	+	+	+	+	+	+
**ATP Binding Family**											
Lactose/L-arabinose	−	−	−	−	−	−	+	+	+	+	+
Maltose/Maltodextrin	+	+	+	+	+	+	+	+	+	+	+
Methyl-Galactoside	−	+	−	−	−	−	−	−	−	−	−
Multiple Sugar (unspecified)	+	+	+	+	+	+	+	+	+	+	+
Oligogalacturonide	−	−	−	−	−	−	+	−	+	−	−
Putative Multiple Sugar (unspecified)	−	−	−	+	+	+	−	−	+	+	+
Putative Sugar (unspecified)	−	−	−	−	−	−	+	−	−	−	−
Ribose	+	+	+	+	+	+	+	+	−	+	+
Simple Sugar (unspecified)	+	+	+	+	+	+	+	+	+	+	+
Xylose	−	+	−	+	+	+	+	+	+	+	+
**PTS Family**											
Cellobiose	+	+	+	+	+	+	+	+	+	+	+
Fructose	+	+	+	+	+	+	+	−	+	+	+
Galactitol	+	+	+	+	+	+	+	+	+	+	+
Galactosamine	−	−	−	−	−	−	−	−	+	+	+
Glucitol/Sorbitol	−	−	−	−	−	−	−	−	+	+	+
Mannitol	+	+	+	+	+	+	+	+	+	+	+
Mannose	+	+	+	+	+	+	+	+	+	+	+
N-Acetylglucosamine	+	+	+	+	+	+	+	+	+	+	+
Sucrose	−	−	−	+	+	+	−	−	+	+	−

Symbols denote the presence (+) or absence (−) of a particular annotated transporter within each genome. Substrate specificity is inferred based upon KO annotated specificity of the substrate binding protein (ABC transporters) or the membrane linked EIIC components (PTS transporters).

1 Transport systems presented are limited to complexes showing co-localization of all genes needed to form a functional complex. Complexes lacking annotation of a single component are not included. Redundancy in transport systems exists, but is not identified.

#### ABC-type transporters

Despite the presence of annotated poly- and oligosaccharide transporters within *Thermoanaerobacter* genomes (Table 2), few studies have investigated the ability of *Thermoanaerobacter* strains to utilize these saccharides. Working with *Thermoanaerobacter* strains not analyzed here, the study by Wiegel *et al*. [64] noted strain specific differences in xylo-oligomer utilization patterns and rates, yet to date, no correlation between genome content and poly- or oligosaccharide utilization has been established for any strain within the genus. The prediction that most *Thermoanaerobacter* CAZymes are intracellular also suggests that these strains have the innate capacity to transport complex soluble carbohydrates as there is little evolutionary advantage to maintain intracellular CAZymes if the saccharides upon which they act cannot be transported into the cell. However, experimental characterization of *Thermoanaerobacter* transport systems is needed. This is particularly true given that the mean degree of polymerization for cellulose hydrolysis products generated by *C. thermocellum* is four [65], and that *C. thermocellum* xylan hydrolysis yields principally xylo-oligomers [66]–[69]. The ability, or lack thereof, to transport, poly- and oligosaccharides in a *Thermoanaerobacter* strain may significantly impact carbohydrate utilization in a co-culture with *C. thermocellum*.

Xylose transport has been one of the most thoroughly investigated sugar import mechanisms within the genus and an ABC-type xylose binding protein was first identified in *T. pseudethanolicus* 39E by Erbeznik *et al*. [70] and a *xylFGH* operon, coding for the entire ABC-type transporter, was later determined in the same strain [71]. However, the amino acid sequences reported do not agree with those identified in the *T. pseudethanolicus* 39E genome. Nevertheless, the reported partial *xylF* sequence and complete *xylGH* sequences reported do show 99.5%, 100% and 100% sequence identity, respectively, and similar genomic architecture, with the annotated xylose ABC-transport system in *T. italicus* Ab9.

In *T. italicus* Ab9, as well as *T. mathranii* subsp. *mathranii* A3, *T. thermohydrosulfuricus* WC1 and *T. wiegelii* Rt8.B1, the annotated xylose transport genes are not co-localized with the xylose isomerase and xylulokinase (*xylAB*) operon. They are however, co-localized in *Thermoanaerobacter* spp. X513, X514 and X561, *T. ethanolicus* CCSD1 and *T. siderophilus* SR4 as is reported elsewhere [23]. *Thermoanaerobacter* spp. X513, X514, and X561, as well as *T. siderophilus* SR4, all contain a secondary gene cluster with an annotated xylose binding protein located further downstream. Recently, Lin *et al*. [72] report that in *Thermoanaerobacter* sp. X514, the xylose transport genes found co-localized with the *xylAB* operon are induced when grown on xylose giving support to the annotation of these genes as xylose transporters. The response of the secondary gene cluster was not discussed and thus, its role in xylose transport is not yet confirmed. The presence of xylose specific ABC-transport systems in some, but not all, *Thermoanaerobacter* strains (Table 2) has been proposed to potentially account for increased xylose uptake and utilization [23]. Assuming this translates throughout the genus, only two strains, *T. brockii* subsp. *finnii* Ako-1 and *T. pseudethanolicus* 39E, may be limited in xylose uptake efficiency based on their annotated genomes, as they may rely on non-specific mechanisms for xylose transport.

The transport of cellobiose and/or glucose has also been proposed to occur via an ABC-type transport system designated *cglF*-*cglG*(*xarG*) with the substrate specificity inferred based upon its proximal location to a glycoside hydrolase (*cglT* – discussed above) showing high activity towards cellobiose in *Thermoanaerobacter brockii* subsp. *brockii* HTD4 [56]. However, Mai *et al*. [55] note that in *T. ethanolicus* JW200 the orthologous gene is co-localized with a bifunctional xylosidase-arabinosidase (*xarB*/*xglS*) and not with a *cglT* ortholog. Thus, it may be involved with transporting xylan hydrolysis products. Only six *Thermoanaerobacter* genomes, *T. brockii* subsp. *finnii* Ako-1, *T. pseudethanolicus* 39E, *T. italicus* Ab9, *T. mathranii* subsp. *mathranii* A3, *T. thermohydrosulfuricus* WC1 and *T. wiegelii* Rt8.B1, contain the *cglF*-*cglG*/*xarG* gene cluster and in all cases, show similar genomic organization to *T. ethanolicus* JW200.

#### PTS-mediated transport

Genes annotated to transport nine different substrates via PTS-mediated mechanisms are identified within the genus (Table 2), though none of these gene sequences have been functionally characterized. Alternative to the ABC-dependent glucose uptake proposed (above), Lin *et al*. [72] have identified genes in *Thermoanaerobacter* sp. X514 which may form a glucose specific PTS complex. Expression analysis of the gene cluster identified (Teth514_0412-Teth514_0414) was specifically linked to mid-exponential phase growth of cells grown on glucose only. However, the KO annotation (KO:K02803/KO:K02804) suggests that this gene cluster is specific for N-Acetyl-glucosamine (GlcNAc) rather than glucose. The KO annotation is further supported by the fact that the phosphotransferase specificity of the orthologous EIICB gene in *Caldanaerobacter subterraneus* subsp *tengcongensis* MB4 (87.7% shared amino acid identity with *Thermoanaerobacter* sp. X514) has a *V_max_* 4-fold higher for GlcNAc than it does for glucose [73]. Additionally, the studies by Ng and Zeikus [74] on *T. pseudethanolicus* 39E and Cook *et al*. [75] on *T. wiegelii* Rt8.B1 have identified that glucose import is via non-PTS-mediated transporters. Thus, the genes responsible for glucose uptake in any *Thermoanaerobacter* strain are not yet confirmed.

#### Cationic symporters

Only two distinct carbohydrate-relevant types of cationic symporters are annotated within the genus (Table 2). The study by Hemme *et al*. [23] has proposed that Na^+^ gradient-linked transport may be a mechanism for xylose uptake in *T. pseudethanolicus* 39E, which lacks an annotated xylose specific ABC-type transport system (above). While the substrate binding specificity of the annotated cationic symporters is not yet known, experimental characterization of these enzymes may shed valuable insights into *Thermoanaerobacter* spp. carbon transport, particularly in strains lacking annotated carbon-specific transport systems.

### Carbohydrate Utilization

While cellulose is comparatively chemically homogenous (β-1,4-glucose linkages), hemicellulose fractions are chemically and structurally diverse. Thus, the products of lignocellulose hydrolysis will generate a mixed pool of saccharides available for utilization. While sugar composition ratios and linkages vary from hemicellulose to hemicellulose, the principal simple saccharides of hemicellulose are xylose, arabinose, mannose, glucose, galactose and glucuronic acid [76]–[78]. As the conversion of hemicellulose to ethanol plays an important role in making lignocellulosic biofuels economically feasible [79], [80], identifying a strain capable of fermenting multiple sugars, particularly multiple sugars simultaneously, into ethanol is essential.

#### Utilization of hexose sugars

The potential for all sequenced strains within the genus to utilize the seven primary constituent sugars of lignocellulose was evaluated (Table 3, Table S3). All strains contain a complete Embden-Meyerhoff-Parnas (EMP) pathway. Redundancy in genome annotations for the EMP pathway were only observed for genes encoding glucokinase, 6-phopshofructokinase, fructose-1,6-bisphosphate aldolase and phosphoglyceromutase (Table S3).

**Table 3:** Identification of the genomic potential for sequenced *Thermoanaerobacter* strains to utilize the major carbohydrate hydrolysis products of lignocellulose degradation.

			Clade 1						Clade 2		Clade 3		
			*T. brockii* subsp. *finnii* Ako-1	*T. ethanolicus* CCSD1	*T. pseudethanolicus* 39E	*Thermoanaerobacter* sp. X513	*Thermoanaerobacter* sp. X514	*Thermoanaerobacter* sp. X561	*T. italicus* Ab9	*T. mathranii* subsp. *mathranii* A3	*T. siderophilus* SR4	*T. thermohydrosulfuricus* WC1	*T. wiegelii* Rt8.B1
Cellulose Hydrolysis Products^1^	Glucose	Genes Present	+	+	+	+	+	+	+	+	+	+	+
		Reported Phenotype	+	NR	+	+	+	+	+	+	+	+	+
	Cellobiose	Genes Present	+	+	+	+	+	+	+	+	+	+	+
		Reported Phenotype	+	NR	+	NR	NR	NR	+	+	+	+	+
Hemicellulose Hydrolysis Products	Arabinose	Genes Present	−	−	−	−	−	−	+	+	−	−	−
		Reported Phenotype	−	NR	–	NR	NR	NR	+	+	−	−	−
	Galactose	Genes Present	+	+	+	+	+	+	+	+	+	+	+
		Reported Phenotype	+	NR	+	NR	NR	NR	+	−	NR	+	+
	Mannose	Genes Present	+	+	+	+	+	+	+	+	+	+	+
		Reported Phenotype	+	NR	+	NR	NR	NR	+	+	NR	+	+
	Xylose	Genes Present	+	+	+	+	+	+	+	+	+	+	+
		Reported Phenotype	+	NR	+	+	+	+	+	+	+	+	+
	Glucuronic Acid	Genes Present	−	−	−	−	−	−	+	+	−	+	−
		Reported Phenotype	NR	NR	NR	NR	NR	NR	NR	NR	NR	NR	NR
Reference			[83]	−2	[14]	[85]	[85]	[85]	[12]	[13]	[54]	[15]	[84]

Symbols denote the presence (+) or absence (–) of all enzymes needed for hydrolysis to pyruvate or whether the substrate has been reported to be used (+) or not used (−) in the literature. NR denotes substrate utilization has not yet been reported for a particular strain.

1 Cellulose hydrolysis products have been limited to glucose and cellobiose and not higher order cellodextrins.

2 Physiological data has not yet been reported for this strain.

Genes for a complete Entner-Doudoroff pathway could not be identified in agreement with previous findings [23]. Additionally, a 6-phosphogluconolactonase encoding gene, potentially allowing for hexose utilization through the oxidative pentose phosphate pathway, was not identified in any of the genomes. This pathway has been shown to be non-functional in *Thermoanaerobacter* sp. X514 [81]. However, genes encoding enzymes common to both the Entner-Duodoroff and pentose phosphate pathways are identified within the genomes as they may serve as entry points into the EMP pathway for the catabolism of specific sugars.

All of the sequenced genomes contain a mannose-6-P isomerase responsible for converting mannose-6-P to fructose-6-P and needed for mannose utilization. Additionally, a conserved 5 gene cluster needed for conversion of galactose to glucose-6-P via the Leloir pathway was identified in all of the genomes. This cluster is orthologous to the novel *gal* operon characterized in *Ca. subterraneus* subsp. *tengcongensis* MB4 [82].

The entry points into the EMP pathway for the products of cellobiose hydrolysis are somewhat difficult to predict and would largely be dependent on the mode of transport into the cell (see above), which is not yet known. Transport via an ABC-type system would import cellobiose, while transport via a PTS transporter would yield cellobiose-6-P. In the former situation, cellobiose could be hydrolyzed to two glucose molecules or, if hydrolyzed using a cellobiose phosphorylase (Table S3), could yield 1 glucose +1 glucose-1-P using an inorganic phosphate for phosphorylation. If cellobiose import is via a PTS transporter, hydrolysis would yield 1 glucose +1 glucose-6-P and could occur via any of the GH1, GH3, GH4 or GH5 enzymes identified in all *Thermoanaerobacter* strains (Table S2, Table S3). To date, only the *cglT* gene characterized in *T. brockii* subsp. *brockii* HTD4, which has homologs in all other *Thermoanaerobacter* genomes (see above), has been shown to have β-glucosidase (including cellobiose) activity [56]. Transformation from glucose-1-P to glucose-6-P could occur via a phosphoglucomutase (COG0637; KO:K01838) found in all genomes.

Genes for glucuronic acid metabolism are not universally conserved throughout the genus. Only the Clade 2 strains, plus *T. thermohydrosulfuricus* WC1, have the necessary genes for conversion of glucuronic acid to glyceraldehyde-3-P+pyruvate in conserved 5-gene clusters. Multiple genes are annotated to encode for a 2-keto-3-deoxyphosphogluconate (KDPG) aldolase (COG0800; KO:K01625; TIGR01182) in all *Thermoanaerobacter* strains, though. This enzyme, which is common to the Entner-Doudoroff pathway, also serves as the entry point in glucuronic acid utilization into the EMP pathway. However, with the exception of the strains mentioned above, the necessary genes for glucuronic acid utilization are not identified and thus the role of these annotated KDPG aldolases is difficult to infer.

#### Utilization of pentose sugars

The present analysis confirms that in all strains, xylose is likely isomerized and phosphorylated via xylose-isomerase (*xylA*) and xylulose-kinase (*xylB*) reactions prior to entering the pentose phosphate pathway, in agreement with previous findings [23]. Arabinose utilization genes seem to be limited to Clade 2 strains. Both *T. italicus* Ab9 and *T. mathranii* subsp. *mathranii* A3 have a conserved 3-gene cluster (Table S3) annotated as L-arabinose-isomerase, L-ribulokinase and L-ribulose-5-P-4-epimerase needed to convert L-arabinose to D-xylulose-5-P prior to entering the pentose phosphate pathway.

Of the 77 *in silico* predictions for carbohydrate utilization (11 genomes x 7 carbohydrates), 45 agree with phenotypes reported in the literature [12]–[15], [54], [83]–[85] (Table 3). Thirty-one of the strain-substrate combinations have not yet been investigated experimentally to either confirm or refute these predictions and one prediction (galactose utilization by *T. mathranii* subsp. *mathranii* A3) disagrees with reported phenotypes [13]. *T. mathranii* subsp. *mathranii* A3 has orthologs to the functionally characterized *gal* operon (mentioned above) in *Ca. subterraneus* subsp. *tengcongensis* MB4. The arrangement of the genes is identical to *Ca. subterraneus* subsp. *tengcongensis* MB4 and the annotated genes share >89% amino acid sequence similarity to each respective ortholog. Thus, the reason galactose utilization was not observed by Larsen and coworkers [13] may be due to regulatory differences, a few select mutations affecting enzyme functionality or even an inability to transport galactose, but the exact reason is not clear at this time.

Only Clade 2 strains have the potential to utilize all of the major lignocellulose hydrolysis products (Table 3). However, the significance of this in CBP terms may vary dependent on the nature of the lignocellulosic feedstock. As the composition of hemicellulose varies between feedstocks, the inability to utilize substrates in low abundance may represent acceptable losses for any single CBP system. Alternatively, using a strain with diverse substrate utilization capabilities affords flexibility in designing a CBP system, independent of the nature of the biomass feedstock, not present in strains lacking the ability to utilize specific hemicellulose-relevant saccharides.

### Pyruvate Catabolism and End-product Synthesis

#### Pyruvate catabolism

Fermentation of the above mentioned carbohydrates leads to the formation of pyruvate (Figure 1), a key branch point in *Thermoanaerobacter* carbohydrate metabolism. All *Thermoanaerobacter* strains are reported to have branched catabolic pathways from pyruvate, which yield both ethanol and non-ethanol end-products in varying end-product ratios (Table 4). As such, understanding pyruvate catabolism, and identifying mechanisms to maximize carbon flow towards ethanol, is an important component in making *Thermoanaerobacter* strains industrially relevant.

**Table 4:** Reported end-product yields and related growth conditions for sequenced *Thermoanaerobacter* strains^1^ grown on glucose, xylose or cellobiose.

		End Products (mol/mol hexose equivalent)					Culture Conditions			
Clade	Strain	H_2_	CO_2_	Acetate	Ethanol	Lactate	Substrate	Concentration	Type	Reference
1	*T. brockii* subsp. *finnii* Ako-1	0.25	1.66	0.26	1.66	0.38	Glucose	5 g L^−1^	B	[121]
	*T. pseudethanolicus* 39E	NI	NQ	0.38	1.11	0.30	Glucose	NR	B	[23]
		NI	NQ	0.29	1.52	0.10	Xylose	2 g L^−1^	B	[23]
		0.45	1.72	0.10	1.41	0.15	Glucose	10 g L^−1^	B	[90]
		NI	1.63	0.08	1.55	0.23	Glucose	4 g L^−1^	B	[122]
		NI	NI	0.24	1.25	0.32	Xylose	5 g L^−1^	B	[123]
		NI	NI	0.17	1.10	0.35	Glucose	5 g L^−1^	B	[123]
	*Thermoanaerobacter* sp. X513	NI	NI	NI	NQ	NI	Xylose	1.5 g L^−1^	B	[85]
	*Thermoanaerobacter* sp. X514	NI	NQ	0.51	1.02	0.29	Glucose	NR	B	[23]
		NI	NQ	0.25	1.55	0.12	Xylose	2 g L^−1^	B	[23]
		NI	NQ	0.33	1.25	0.17	Glucose	2.1 g L^−1^	B	[81] 2
	*Thermoanaerobacter* sp. X561	NI	NI	NI	NQ	NI	Xylose	1.5 g L^−1^	B	[85]
2	*T. italicus* Ab9	NQ	NC	NC	NC	NC	Glucose	5 g L^−1^	B	[12] 3
	*T. mathranii* subsp. *mathranii* A3	1.08	2.17	0.48	1.32	0.07	Xylose	2 g L^−1^	B	[13]
3	*T. siderophilus* SR4	NQ	NQ	NI	NQ	NQ	Glucose	5 g L^−1^	B	[54]
	*T. thermohydrosulfuricus* WC1	0.56	1.19	0.71	0.61	0.33	Cellobiose	2 g L^−1^	B	[15]
	*T. wiegelii* Rt8.B1	NI	NI	0.50	0.88	0.17	Glucose	8.6 g L^−1^	B	[109] 2
		NI	1.60	0.50	1.10	0.04	Glucose	9 g L^−1^	P	[124] 4
		NI	1.84	0.53	1.32	0.06	Xylose	7.5 g L^−1^	P	[124] 4
		NI	1.68	0.61	1.06	ND	Cellobiose	17 g L^−1^	P	[124] 4

Abbreviations: NI = end-product not investigated; ND = end-product investigated, but not detected; NQ = end-product detected, but not quantified; NC = end-product quantified, but ratio not calculable due to missing substrate consumption data; B = batch culture; P = pH-controlled batch culture.

1 *T. ethanolicus* CCSD1 omitted as no physiological data is available.

2 End product and substrate utilization data used for calculations approximated from graphical data.

3 Succinate production reported as a minor product.

4 Propionate detected on xylose grown cells (molar ratio = 0.12) and cellobiose grown cells (molar ratio = 0.06).

Pyruvate decarboxylation in all *Thermoanaerobacter* strains, forming acetyl-CoA+CO_2_+ reducing equivalents, appears to proceed through the use of pyruvate:ferredoxin oxidoreductase (POR). This is supported by the identification of genes (Table S4) homologous to the single subunit characterized POR in the phylogenetically related *Moorella thermoacetica* [86], [87] and that multiple investigations of Clade 1 strains have reported a significant role for Fd, as well as ferredoxin:NAD(P)H reductase activity [88]–[90]. Four-gene clusters, annotated as the alpha, beta, gamma and delta subunits of a multi-subunit POR complex are also identified within all strains (Table S4). It is difficult to predict on an *in silico* basis which gene or gene clusters encode the primary POR responsible for pyruvate catabolism and which encode gene products that may act on alternative keto-acids such as indolepyruvate, 2-ketoisovalerate or 2-ketoglutarate.

#### Lactate synthesis

The production of lactate has been reported for all *Thermoanaerobacter* strains with physiological data available (Table 4) and occurs via the reduction of pyruvate using a lactate dehydrogenase (*ldh*) enzyme. Strains in all 3 clades have a single gene annotated as a *ldh* (KO:K00016; TIGR01771), though by COG annotation (COG0039), these same genes are designated as malate/lactate dehydrogenases. Distinguishing between *ldh* and malate dehydrogenase (*mdh*) genes *in silico* can be difficult, though the CDS identified in all genomes (Table S4), with the exception of *Thermoanaerobacter* sp. X561 (truncated due to contig break), share >86% amino acid sequence similarity with the characterized *ldh* from *Thermoanaerobacterium saccharolyticum* [91]. As no other obvious *ldh* is identified, and lactate production is reported throughout the genus, the genes identified in Table S4 are proposed to catalyze *Thermoanaerobacter* lactate formation.

#### Acetate synthesis

Strains of *Thermoanaerobacter* are also reported to produce acetate (Table 4). POR mediated pyruvate catabolism will yield acetyl-CoA, which can be converted to acetate +1 ATP via phosphotransacetylase (*pta*) and acetate kinase (*ack*). In all strains, the PTA and ACK enzymes are co-localized within the genome (Table S4). Three strains, *Thermoanaerobacter* spp. X513, X514 and X561 have additional *ack* genes annotated, though these are not co-localized with *pta* genes. Working with a non-sequenced *Thermoanaerobacter* strain, *Thermoanaerobacter thermohydrosulfuricus* DSM570, Mayer *et al*. [92] observed a severe reduction in acetate production and enzyme activity in *pta* ^−^ and *ack* ^−^ mutants and additionally proposed that the *pta* and *ack* genes were co-localized and formed an operon. The residual acetate production observed may be in part due to the fact that in all sequenced strains, additional gene sequences annotated as phosphate butyryltransferases and butyrate kinase genes are also identified (Table S4), and the substrate specificity of these genes is not yet known.

#### Ethanol synthesis

Ethanol production occurs via the reduction of acetyl-CoA to acetaldehyde via an acetaldehyde dehydrogenase followed by a second reduction to ethanol via an alcohol dehydrogenase. Three functionally characterized alcohol dehydrogenase (ADH) genes, *adhA*, *adhB* and *adhE*, have been reported to be principally involved with ethanol formation and a model describing the physiological roles of each gene has been proposed [92]. The *adhA* gene from *T. ethanolicus* JW200 is a reported Zn-binding NADPH-dependent primary alcohol dehydrogenase [94], [95]. In comparison, the *adhA* gene from *T. pseudethanolicus* 39E, which is capable of utilizing both NADH and NADPH, showed a higher catalytic efficiency for NADH oxidation over NADPH oxidation [96]. Pei *et al*. [93] demonstrated, *in vitro*, that the *adhB* and *adhE* gene products from *T. ethanolicus* JW200 displayed bifunctional acetaldehyde/alcohol dehydrogenase activity despite the fact that only the *adhE* gene contained two independent domains related to aldehyde dehydrogenase and alcohol dehydrogenase families, respectively. However, when assayed using measured intracellular concentrations of NAD(P)^+^ and NAD(P)H, the *adhE* gene product displayed only aldehyde dehydrogenase activity (NADH dependent), while the *adhB* gene strongly favored acetaldehyde reduction over acetyl-CoA reduction.

Only five *Thermoanaerobacter* genomes contain genes annotated as standalone aldehyde dehydrogenases (Table S4), but no evidence yet exists to suggest that these genes function as acetaldehyde dehydrogenases. Furthermore, genomic context provides no further insights into substrate specificity. As such, it is likely that the reduction of acetyl-CoA to acetaldehyde via *adhE* is a conserved physiological process throughout the genus. The *adhB* encoding gene in *T. pseudethanolicus* 39E is considered to be NADPH-dependent [97] and, upon ethanol accumulation, has shown a higher specific activity towards ethanol oxidation as opposed to ethanol formation [93]. Thus, its role *in vivo* is not yet confirmed.

The three ADH genes, *adhA, adhB* and *adhE*, in conjunction with a recently described redox-sensing transcriptional regulator in *T. ethanolicus* JW200 [98], have largely formed the basis for our understanding of *Thermoanaerobacter* ethanologenesis in a few select strains. However, given that the sequenced *Thermoanaerobacter* genomes have annotated anywhere from 5–9 putative alcohol dehydrogenases, most of which have unknown specificity, this model of ethanol metabolism may not fully encompass all ethanol producing reactions within the cell.

**Figure 3:**
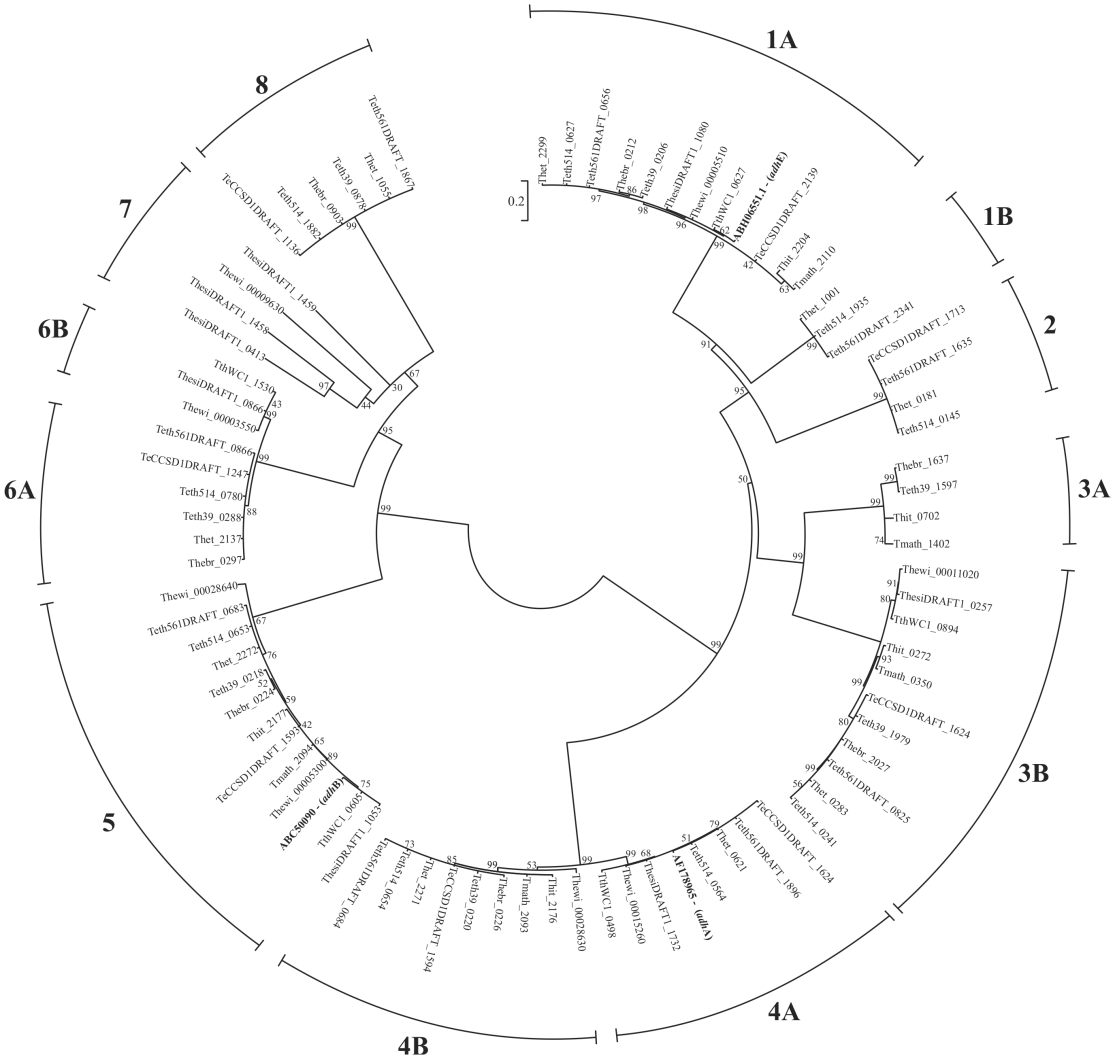
Phylogenetic analysis of all annotated alcohol dehydrogenase genes within sequenced *Thermoanaerobacter* strains. All included sequences belong to COG1063, COG1454 or COG1979. Tmath_0755 was excluded from analysis as the annotated sequence appears to be a CDS fragment. Sequences in bold correspond to the GenBank accession numbers for functionally characterized sequences from *T. ethanolicus* JW200 [93], [95]. Tree construction was as described in Materials and Methods. Bootstrapping support values are indicated by their respective nodes.

The three characterized *Thermoanaerobacter* alcohol dehydrogenase genes belong to COG1454-Class IV alcohol dehydrogenase (*adhA*, *adhE*) and COG1063-Threonine dehydrogenase and related Zn-dependent dehydrogenases (*adhB*). Additional sequences belonging to each COG designation were identified, as well as sequences belonging to COG1979-Uncharacterized oxidoreductase, Fe-dependent alcohol dehydrogenase family (Table S4). To identify whether the genomes encode potential additional ethanol producing alcohol dehydrogenases, we conducted phylogenetic analysis of all gene sequences identified in COG1063, COG1454 and COG1979 (Figure 3) as a means of inferring specificity.

Of the 80 gene sequences analyzed, 8 distinct clades, and an additional 4 sub-clades were identified. Gene sequences homologous to *adhA* were identified in Clusters 4A and 4B. Additionally, 5 genomes contained paralogous pairs within these clusters that showed >90.3% amino acid sequence identity within each respective pair. Microarray data from Hemme *et al*. [23] have shown that in *Thermoanaerobacter* sp. X514, both genes in the paralagous pair (Teth514_0564– Cluster 4A; Teth514_0654– Cluster 4B) are expressed. Additionally, in one case (Teth514_0564), expression is dependent on growth conditions. All genomes contained sequences orthologous to the characterized *adhB* gene (Cluster 5) as well as the *adhE* gene (Cluster 1A). Three strains, *Thermoanaerobacter* spp. X513, X514 and X561 contained an additional *adh* gene that grouped near the *adhE* orthologs (Cluster 1B), though the annotation of these sequences suggests only alcohol dehydrogenase, and not aldehyde dehydrogenase, activity. Surprisingly, Clusters 3A and 3B, which belong to COG1979, group more closely to Clusters 1 and 2 (COG1454) than does Cluster 4 (also COG1454).

Given the number and diversity of *adh* genes annotated, and that expression patterns of homologous genes in different strains are distinct [23] suggesting differential regulation, the current 3-gene model [93] proposed for ethanol formation in *Thermoanaerobacter* spp. may not translate across all strains. This is supported by the fact that the study by Hemme *et al*. [23] identified varied expression of 8 of the 9 *adh* annotated sequences in *Thermoanaerobacter* sp. X514 under different growth conditions. However, given that multiple *Thermoanaerobacter* spp. have been reported to grow on sugar alcohols [12], [13], [16], [58], [84], it is possible that some of these may have catabolic functions and are not involved with ethanol synthesis. Thus, understanding *Thermoanaerobacter* ethanologenesis requires more in depth functional and expression analysis studies across multiple strains.

#### Hydrogen synthesis

Hydrogen production, similar to lactate production, competes for reducing equivalents with ethanol synthesis to a greater or lesser extent in all strains of *Thermoanaerobacter* (Table 4), but in-depth cross-species analysis of *Thermoanaerobacter* hydrogenases has yet to be conducted.

#### [Fe-Fe] hydrogenases

Gene sequences homologous to the cytosolic NADH-dependent Fe-only enzyme characterized in *Ca. subterraneus* subsp. *tengcongensis* MB4 [99] are conserved in all strains of the *Thermoanaerobacter* genus (Table 5). Sequence analysis of the hydrogenase conserved domains suggests that these hydrogenases are most similar to the heterotrimeric A1 group exhibiting a TR(M3) modular structure described by Calusinska *et al*. [45]. These gene products are thought to be NAD-dependent due to the presence of NADH-binding domains in the accessory subunits. However, in *Ca. subterraneus* subsp. *tengcongensis* MB4, these same genes, as well as the orthologs in *T. pseudethanolicus* 39E have recently been proposed to function as potential bifurcating [Fe-Fe] hydrogenases [100], which couple the thermodynamically unfavourable oxidation of NADH to H_2_ production through utilization of the exergonic oxidation of Fd_red_.

**Table 5:** Annotated hydrogenases within sequenced *Thermoanaerobacter* spp.

		FeFe hydrogenases			Ni-Fe hydrogenase
		Bifurcating	Fd-linked	PAS-sensory	Ech
**Clade 1**	*T. brockii* subsp. *finnii* Ako-1	Thebr_1491–Thebr_1495	Thebr_1498	Thebr_0227	
	*T. ethanolicus* CCSD1	TeCCSD1DRAFT_0391–TeCCSD1DRAFT_0395	TeCCSD1DRAFT_0398	TeCCSD1DRAFT_1595	
	*T. pseudethanolicus* 39E	Teth39_1456–Teth39_1460	Teth39_1463	Teth39_0221	
	*Thermoanaerobacter* sp. X513	Thet_0793–Thet_0797	Thet_0790	Thet_2270	
	*Thermoanaerobacter* sp. X514	Teth514_2138–Teth514_2142	Teth514_2145	Teth514_0655	
	*Thermoanaerobacter* sp. X561	Teth561DRAFT_0433–Teth561DRAFT_0437	Teth561DRAFT_0440	Teth561DRAFT_0685	
**Clade 2**	*T. italicus* Ab9	Thit_0826–Thit_0830	Thit_0823	Thit_2175	Thit_1612–Thit_1617
	*T. mathranii* subsp. *mathranii* A3	Tmath_0865–Tmath_0869	Tmath_0862	Tmath_2092	Tmath_1603–Tmath_1608
			Tmath_1048		
**Clade 3**	*T. siderophilus* SR4	ThesiDRAFT1_1833–ThesiDRAFT_1837	ThesiDRAFT1_1830		ThesiDRAFT1_1402–ThesiDRAFT1_1407
	*T. thermohydrosulfuricus* WC1	TthWC1_1780–TthWC1_1784	TthWC1_1787		TthWC1_1092–TthWC1_1097
	*T. wiegelii* Rt8.B1	Thewi_00017870–Thewi_00017920	Thewi_00017850	Thewi_00028620	Thewi_00009040–Thewi_00009090
			Thewi_00028740	Thewi_00005270	

Upstream of the genes in the A1 grouping in all *Thermoanaerobacter* strains is a histidine kinase protein as well as another putative hydrogenase gene which shows similar domain architecture to group D hydrogenases. This is consistent with the genomic organization described for bifurcating hydrogenases [45], which is also observed in *Ca. subterraneus* subsp. *tengcongensis* [99].

PAS-domain (pfam00989) containing sensory hydrogenases are also conserved throughout the genus with the exception of *T. siderophilus* SR4 and *T. thermohydrosulfuricus* WC1 (Table 5). Sensory hydrogenases have been reported to be linked with histidine kinase based signal transduction mechanisms and could play a role in regulating cellular redox levels [45], [101]. *T. wiegelii* Rt8.B1 and *T. mathranii* subsp. *mathranii* A3 both contain additional [Fe-Fe]-hydrogenases showing modular structures similar to the B1 or B3 monomeric hydrogenases described by Calusinska *et al*. [45].

#### [Ni-Fe] hydrogenases

[Ni-Fe] hydrogenase encoding genes can be identified for strains belonging to Clade 2 and Clade 3, but not to strains in Clade 1 (Table 5). Strains in Clades 2 and 3 both have the conserved 6-gene cluster coding for a membrane-bound cation transporting Fd-consuming energy conserving hydrogenase (Ech) directly followed by the 6-gene *hypABFCDE* gene cluster responsible for assembly of the [Ni-Fe] center. Fd_red_ generated via POR in pyruvate catabolism is thought to provide the electrons needed for the evolution of H_2_ using the Ech complex.

Fd-dependence has been shown for the orthologous gene sequences in *Ca. subterraneus* subsp *tengcongensis* MB4 [99]. Furthermore, cell extracts of *Ca. subterraneus* subsp. *tengcongensis* MB4 were shown to favour H_2_-evolution over H_2_-consumption and Ech was additionally proposed to play a role in “proton respiration” [99]. However, to date, the physiological role of Ech has not been determined for any Clostridia and the exact nature of the exported cation (H^+^ of Na^+^) has not yet been determined. It is interesting to note that the absence of Ech encoding homologs (Table 5) in *T. pseudethanolicus* 39E (Clade 1) correlates with the lowest reported molar H_2_ yield (and highest molar ethanol yield) of strains with data available (Table 4). This is perhaps indicative of an important physiological role for Ech in Clade 2 and/or Clade 3 strains. Apart from Ech, no other [Ni-Fe] hydrogenases are identified within any *Thermoanaerobacter* spp. genome.

### Energy Metabolism

Energy metabolism is interconnected with carbon and electron flux and governs many of the physiological processes involved with lignocellulosic ethanol production (Figure 1). The principal forms of metabolic energy in bacteria include ion motive force, ATP and/or in some cases, pyrophosphate (PPi). Understanding inter-strain differences in energy metabolism may help provide insight into the mechanisms governing observed physiological differences (Table 4).

#### Transmembrane ion gradient generating/consuming reactions

**Figure 4:**
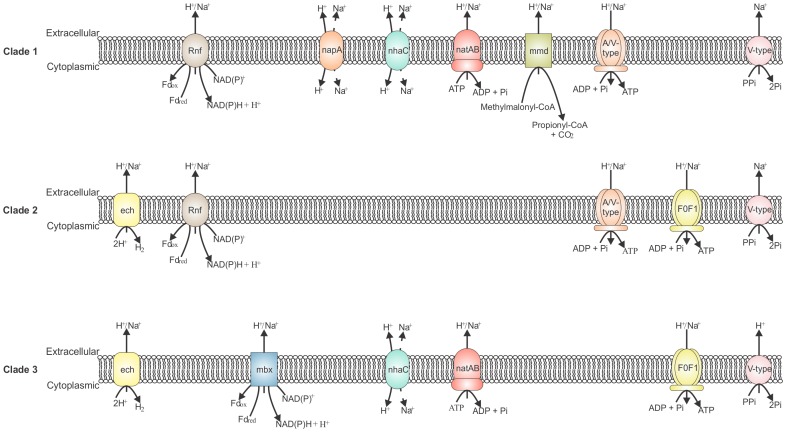
Transmembrane ion gradient generating and consuming reactions involved with *Thermoanaerobacter* cellular energetics. With the exception of the natAB complex (see text, Table S5), all enzyme complexes show intra-clade conservation. Cation specificity is not inferred unless specifically discussed within the text. Dashed lines associated with napA and nhaC antiporters indicate a counter-directional flow of Na^+^ ions in relation to H^+^.

Transmembrane ion gradients can be used to drive endergonic reactions such as solute transport, including carbohydrate transport (Figure 1, Table 2), and ATP synthesis. The mechanisms of balancing ion motive force with the other energy currencies are poorly characterized in *Thermoanaerobacter* spp., but the present *in silico* analysis indicates that the potential energy conserving mechanisms within the genus show significant intra-clade conservation (Figure 4).

Strains of Clade 3 contain a 13-gene cluster (Figure 4) similar in genomic structure to the *mbx* genes of *Pyrococcus furiosus* proposed to encode for a complex with Fd_red_:NAD(P)^+^ oxidoreductase activity [102], with energy released via the oxidation of Fd_red_ being conserved via translocation of a cation. In *P. furiosus,* the *mbx* gene cluster has been proposed to play a role in the reduction of elemental sulfur, where it transfers electrons from Fd_red_ to NAD(P)H, which is subsequently oxidized via a NAD(P)H elemental sulfur oxidoreductase [102]. However, the role in sulfur metabolism for *P. furiosus* has been inferred based upon microarray data indicating increased gene expression of the *mbx* gene cluster in response to the addition of elemental sulfur to the growth medium. Assuming the proposed Fd:NAD(P)^+^ oxidoreductase activity of mbx is also observed in the Clade 3 strains, there is no evidence yet that suggests it is connected with sulfur reduction in these *Thermoanaerobacter* strains.

Clade 1 strains contain apparent remnants of the *mbx* gene cluster (Table S5) though not all 13-genes could be identified. Five genes, the *mbxMJKLM* cluster, are not found immediately following orthologs of the *mbxABCDGGHH*’ gene cluster as observed in Clade 3. Thus, Clade 1 strains do not appear to contain the genes necessary for a functional mbx complex. No orthologs were identified in the Clade 2 strains.

A functionally analogous Fd:NAD(P)^+^ oxidoreductase system, the ion-translocating Rnf complex, is present in all Clade 1 and Clade 2 strains (Figure 4, Table S5). The genomic organization is identical to what is reported for *Acetobacterium woodii* and multiple subunits of the *T. pseudethanolicus* 39E complex have been reported to be genetically similar to the partially characterized protein complex in *A. woodii* [103], [104]. In *A. woodii*, the complex is thought to translocate Na^+^ ions, yet definitive proof has not yet been determined.

Na^+^ energetics may play a prominent role in multiple *Thermoanaerobacter* strains. Of the five classes of recognized primary Na^+^ pumps [105], four of the classes are observed within the genus (Table S5). One class, comprised of Na^+^-translocating decarboxylation reactions, is limited to strains belonging to Clade 1. Hemme *et al*. [23] identify both a methylmalonyl-CoA decarboxylase and an oxaloacetate decarboxylase in the genomes of *T. pseudethanolicus* 39E and *Thermoanaerobacter* sp. X514. However, the present analysis is unable to find locus tags supportive of a membrane-associated oxaloacetate decarboxylase complex.

Annotation of the methylmalonyl-CoA decarboxylase encoding genes (Figure 4, Table S5) is supported by the presence of genes annotated to encode methylmalonyl-CoA mutase and methylmalonyl-CoA epimerase immediately adjacent to the methylmalonyl-CoA decarboxylase annotated gene sequences in all Clade 1 genomes. In all sequenced *Thermoanaerobacter* strains, genes annotated as oxaloacetate decarboxylase subunits (α, β, γ) can be identified, though they are never co-localized into a single gene cluster, as is observed with other Clostridia [106], and a functional membrane associated oxaloacetate decarboxylase complex may not exist in any of the *Thermoanaerobacter* strains.

Genes homologous to the *natAB* genes described in *Bacillus subtilis* [107] are identified in multiple *Thermoanaerobacter* strains, but do not show intra-clade conservation (Table S5). A third primary Na^+^ pump includes the Rnf complex (discussed above). The fourth and final class of potential Na^+^ pumps identified in *Thermoanaerobacter* are the V-type inorganic pyrophosphatases (Table S5). All V-type pyrophosphatases identified in Clades 1 and 2 appear to be orthologous to each other. Genes in Clade 3 though, are significantly different than the V-type pyrophosphatases identified in Clades 1 and 2. Key residues, as determined by Luoto *et al.* [108], were identified for all annotated genes via sequence alignments. The sequences present in Clades 1 and 2 share key residues identical to reported K^+^-dependent, Na^+^-exporting V-type pyrophosphatases, while Clade 3 sequences share identical residues with K^+^-independent, H^+^-exporting versions (Table S6).

Given that the cation specificity for any of the above mentioned ion-translocating processes discussed have not yet been determined experimentally for any *Thermoanaerobacter* strain, it is impossible to predict their role. Cook [109] reports inhibited growth by *T. wiegelii* Rt8.B1 in the presence of the Na^+^ ionophore monensin, thus suggesting the importance on maintaining a transmembrane Na^+^ gradient for cell viability. However, genomic analysis identifies that a H^+^ motive force is also expected in *T. wiegelii* Rt8.B1 (eg. V-type pyrophosphatase). Both gradients may exist, and cellular demands are balanced by the use of H^+^/Na^+^ antiporters. Cation exchange antiporters, showing homology to the NhaC family, can be identified though in all strains except Clade 2 (Figure 4). Clade 1 also contains genes homologous to NapA type antiporters. Surprisingly, analysis of the annotated transport systems for Clade 2 strains does not reveal any cation exchangers. Thus, if Clade 2 strains generate both H^+^ and Na^+^ ion-motive forces, it is unclear how they balance these forces.

#### ATP and pyrophosphate (PPi) as energy currencies

Synthesis of ATP as an energy currency, which is closely linked with carbohydrate and pyruvate metabolism (Figure 1), can occur via glycolysis and through the production of acetate via acetate kinase in all *Thermoanaerobacter* strains. According to its annotation, both Clade 1 and Clade 3 genomes also encode an ATP-linked (in contrast to GTP-linked) PEP carboxykinase based on the enzyme commission number assigned to the annotated sequences (EC: 4.1.1.49) [110]. This bidirectional enzyme could either carboxylate PEP yielding 1 ATP+oxaloacetate or decarboxylate oxaloacetate at the expense of ATP. The flux models for *T. pseudethanolicus* 39E and *Thermoanaerobacter* sp. X514 [23] suggest that, in these two strains, oxaloacetate decarboxylation occurs, but it is unknown if this translates universally throughout the genus.

ATP synthesis via ATP synthase genes can occur through two distinct means within *Thermoanaerobacter* spp. Strains of Clade 1 contain a 9-gene cluster designated as an A/V-type ATP synthase (Figure 3, Table S5), while Clade 3 strains contain the F_0_F_1_-ATP synthase in a conserved 8-gene cluster. Strains of Clade 2 contain both the AV-type and the F_0_F_1_-ATP synthase gene clusters in identical genomic organization as Clade 1 or Clade 3, respectively.

PPi as an alternative energy carrier to ATP has been observed during exponential phase for the phylogenetically related strains *M. thermoacetica* [111] and *Cal. saccharolyticus* DSM 8903 [112]. Many of the key genomic elements to potentially generate and utilize PPi as a central energy carrier are similarly identified within all *Thermoanaerobacter* spp. Pyruvate kinase, which is identified in all *Thermoanaerobacter* strains, is typically considered to be responsible for the conversion of phosphoenolpyruvate to pyruvate. However, two distinct pyruvate phosphate dikinase (PPDK) genes (566–567 and 877 amino acids) are also universally conserved throughout the genus (Table S4). The longer of these genes shows >77.7% amino acid identity with the annotated PPDK gene (Csac_1955) from *Cal. saccharolyticus* DSM 8903, whose genome also encodes a pyruvate kinase [106]. In *Cal. saccharolyticus* DSM 8903, the PPDK has been proposed to function in a catabolic role during exponential growth whereby the conversion of PEP to pyruvate is coupled to the conversion of AMP+PPi to ATP+Pi [112]. Its presence in *Thermoanaerobacter* spp. suggests that PPi may also play a role in cellular energetics.

Additionally, sequence alignments (Figure S1) of the annotated 6-phosphofructokinase (PFK) genes (Table S7) identifies that Clade 1 and Clade 3 strains possess one copy of PFK that contains the conserved Asp_104_+ Lys_124_ residues (*Escherichia coli* numbering) associated with PPi-dependence [113] and also found in *Cal. saccharolyticus* DSM 8903 [112]. Energy may additionally be conserved as an ion-motive force through the use of a membrane linked cation-translocating V-type pyrophosphatase (see above). Strains reported to use PPi as an energy currency during exponential growth are expected to have relatively high intracellular PPi/ATP ratios and low cytosolic PPiase activity. While, the intracellular ATP concentrations of exponential *T. wiegelii* Rt8.B1 cells [109] are reportedly higher than those observed for *Cal. saccharolyticus* DSM 8903 [112], PPi levels and PPiase activity has not yet been investigated for any strain of the *Thermoanaerobacter* genus.

### Conclusions

The analyses presented here have identified inter-strain differences at the genomic level within the *Thermoanaerobacter* genus that may account for differences in the industrial application of these bacteria in a CBP system. Based on genomic content, Clade 2 strains seem most well suited to biomass hydrolysis and utilization, though these strains have not yet been reported to have as high of ethanol yields as some Clade 1 strains do (Table 4). Conversely, despite the ethanologenic capabilities of Clade 1 strains, their genomes encode the fewest extracellular CAZymes of all strains within the genus (Table 1), which may potentially limit their hydrolytic capabilities. The genomes of Clade 3 strains show intermediate hydrolytic and substrate utilization capabilities, but also represent the most divergent lineage of the genus (Figure 2) and may have yet unexamined potential.

The use of a specific strain for development of a universal approach to lignocellulosic ethanol production may represent an idealistic concept. Rather, strain selection may be specific to a single CBP system and dependent on the nature of the feedstock. Efficient conversion of arabinose to ethanol is of little value for bioenergy feedstocks such as eucalyptus, which contains comparatively low amounts of arabinan (0.3%), in contrast to switchgrass (3.0%) [114]. Alternatively, extracellular xylan hydrolysis may not be an essential component in the microbial conversion of feedstocks such as softwoods, which have a low xylan and high glucomannan hemicellulose content [76], [115]. This is particularly true given that no extracellular glucomannanases were identified in any strain of *Thermoanaerobacter* (Table 1).

Development of biocatalysts with desired physiological characteristics using a strain with diverse, rather than specialized capabilities may be advantageous for constructing a robust and dynamic CBP system. For example, the construction of a single mutant (Δ*ldh*) in *T. mathranii* BG1, a platform organism of BioGasol (http://www.biogasol.com) [116], has shown to improve ethanol yields and still maintain substrate utilization capabilities similar to *T. mathranii* subsp. *mathranii* A3 (Clade 2). Alternatively, strategies that broaden the capabilities of a relatively specialized strain have also shown to be successful. The cloning and expression of a functional endoglucanase into *Thermoanaerobacter* sp. X514 [117], a comparatively good ethanol producer, has improved that strain’s hydrolytic capabilities.

The purpose of this paper was to evaluate genomic differences within members of the *Thermoanaerobacter* genus which may influence strain suitability in a CBP co-culture system. Also, correlating genome content with the reported physiologies is a first step to help shape molecular engineering strategies for strain improvement. It is important to consider though that the analysis presented here is of the genomic potential of sequenced *Thermoanaerobacter* strains and is not of the observed phenotypes. Future experiments such as expression profiling studies and enzymatic characterization, which can supplement the data presented here, will help to improve our understanding of the extent that the genomic potential is achieved within these strains. Experiments targeted towards improving the hydrolysis of raw lignocellulosic biomass, understanding carbon transport and simultaneous utilization of mono-, oligo-, and polysaccharides, evaluating the genomic and regulatory basis for the observed differences in end-product synthesis ratios and improving the correlation between energy metabolism and end-product yields will all help develop *Thermoanaerobacter* spp. into more efficient CBP microorganisms.

This study is focused on components associated with lignocellulosic biofuel production and does not investigate the genomics associated with other phenotypes reported within the genus such as peptide and amino acid oxidation [118], metal-reduction [54], [85], [119] or sulfur reduction [50]. The potential impact that these phenotypes may have on biofuel production is not yet known. For example, vitamin B_12_ biosynthesis, associated with co-factor metabolism, has recently been shown to play an important role in improving observed ethanol yields [22], [23] in select *Thermoanaerobacter* strains. Therefore, we cannot discount the possibility that additional components of cellular physiology may similarly influence the lignocellulosic ethanol production capabilities of these strains. However, this work does identify key genomic criteria pertinent to strain evaluation for the development of a *C. thermocellum*-*Thermoanaerobacter* sp. co-culture and represents the most comprehensive comparative genomic analysis of the genus to date. Furthermore, comparative genomic analysis such as this can be useful in identifying important physiological questions to address through experimentation not only for *Thermoanaerobacter* spp., but also in other organisms of interest for lignocellulosic ethanol production through CBP.

## Supporting Information

Figure S1 **Partial sequence alignment of selected PFK genes in different bacteria.**
(PDF)

Table S1 **Selected genome metadata for sequenced** ***Thermoanaerobacter*** **spp.**
(XLS)

Table S2 **All CAZyme designated gene sequences within sequenced** ***Thermoanaerobacter*** **strains as are available within the CAZy database or identified through** ***de novo*** **analysis.**
(XLS)

Table S3 **Identified genes associated with the utilization of the major carbohydrates produced through lignocellulose hydrolysis in sequenced** ***Thermoanaerobacter*** **strains.**
(XLS)

Table S4 **Genes associated with pyruvate metabolism in sequenced** ***Thermoanaerobacter*** **strains.**
(XLS)

Table S5 **Genes associated with transmembrane ion gradient generating and consuming reactions involved with cellular energetics in sequenced** ***Thermoanaerobacter*** **genomes.**
(XLS)

Table S6 **Key amino acid residues responsible for imparting predicted substrate specificity and K^+^ dependence in annotated** ***Thermoanaerobacter*** **V-type pyrophosphatases.**
(XLS)

Table S7 **Identification of key residues characteristic of ATP or PPi dependent 6-phosphofructokinase genes in** ***Thermoanaerobacter*** **.**
(XLS)
