# Supplementary material for: Genomic Evaluation of Thermoanaerobacter spp. for the Construction of Designer Co-Cultures to Improve Lignocellulosic Biofuel Production
Source: PLoS One. 2013 Mar 26;8(3):e59362. doi: 10.1371/journal.pone.0059362 (PMC3608648; doi:10.1371/journal.pone.0059362)
Supplement: Figure S1 — Partial sequence alignment of selected PFK genes in different bacteria. (PDF) [file pone.0059362.s001.pdf]

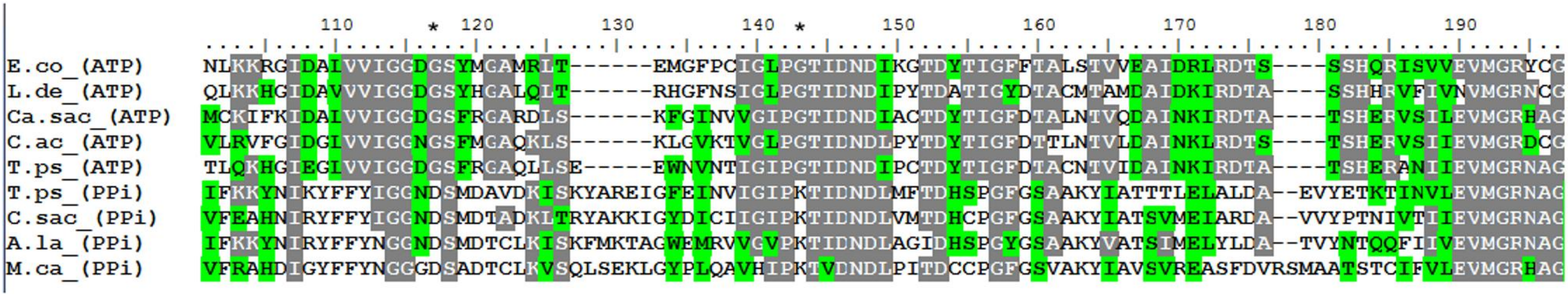

Figure S1. Partial sequence alignment of selected PFK genes in different bacteria. Sequences are similar to those chosen by Bielen *et al*. [112]. The PFK sequences annotated in *T. pseudethanolicus* 39E were chosen as representative examples of PPi and ATP-dependent PFK genes within the genus *Thermoanaerobacter*. Abbreviations are as follows: E.co (*Escherichia coli*), L.de (*Lactobacillus delbrueckii*), C.sa (*Ca. saccharolyticus*), C.ac (*Clostridium acetobutylicum*), *T.ps* (*T. pseudethanolicus* 39E), A.la (*Archoleplasma laidlawii*), M.ca (*Methylococcus capsulatus*). Marked positions (*) are indicative of conserved residues important in determining PPi or ATP dependence. Amino acid signatures for all *Thermoanaerobacter* strains are available in Table S7.
